# The Impact of Technology on Anxiety Management in University Students

**DOI:** 10.3390/bs15030310

**Published:** 2025-03-05

**Authors:** Paula Puente-Torre, Vanesa Delgado-Benito, Sonia Rodríguez-Cano, Miguel Ángel García-Delgado

**Affiliations:** Faculty of Education, Department of Education, University of Burgos, Castilla y Leon, 09001 Burgos, Spain; vdelgado@ubu.es (V.D.-B.); srcano@ubu.es (S.R.-C.); miguelangelgd@ubu.es (M.Á.G.-D.)

**Keywords:** anxiety, college students, state anxiety, trait anxiety

## Abstract

Anxiety is one of the main disorders affecting university students, partly due to the current predominant pace of life and the competitive environment of the university. In this sense, the use of technology to reduce anxiety is one of the main tools available to university students. In order to analyze the current situation of the university population with respect to anxiety levels, both state anxiety and risk anxiety, the State-Trait Anxiety Inventory Questionnaire (STAI) was used and applied to a sample of four hundred and fifty students at the University of Burgos, ensuring the representativeness of this population by means of non-probabilistic sampling. Descriptive research has been carried out to determine the levels of anxiety of students at the University of Burgos. Likewise, the benefits of different studies on the application of technology to reduce anxiety are presented, as well as the applications most used by university students. In general terms, we can consider that the implementation of mindfulness techniques through emerging technologies promotes a reduction in anxiety levels among the university population. However, it is necessary to clarify that the data presented only indicate a correlation, not a relationship of cause and effect. Finally, various proposals for improvement and expansion of the research carried out are offered.

## 1. Introduction

Anxiety is one of the most prevalent psychological illnesses in society worldwide, with 264 million cases of anxiety disorder, equivalent to 3.6% of the world’s population (Kipnis et al., 2016). It is also the sixth leading cause of disability worldwide, with no significant change since 1990 (American Psychiatric Association, 2014).

All individuals experience anxiety at some point in their lives; it is distinguished by a vague and unpleasant feeling of worry, often accompanied by physical symptoms such as headache, excessive sweating, rapid heart rate, tightness in the chest, stomach discomfort, and restlessness. It acts as a warning signal that warns of impending danger and allows the individual to take action to cope with the threat, as evidenced by Kuong and Concha (2017). According to Alonso et al. (2004), two types of anxiety are identified: state anxiety, which is transitory and depends on the individual’s experience at a given moment, and trait anxiety, which refers to permanent anxiety without being associated with a specific reason or moment.

In Spain, 49.2% of patients visiting primary care clinics meet the diagnostic criteria for at least one probable anxiety, depression, or somatization disorder (Baxter et al., 2014). This condition can manifest from mild to severe levels, affecting more women than men globally (Pereira et al., 2016).

Recently, studies such as Amaro et al. (2024) have confirmed that the prevalence of anxiety in countries such as Portugal is similar to that of Spain, with 47.8% of young adults reporting clinically significant anxiety symptoms, which reinforces the need to address this problem from a cross-national perspective.

The university stage represents a period of life in which most students begin to consolidate their life projects, come of age, assume new social responsibilities, and face increased psychosocial pressures, where a combination of factors may increase their vulnerability to psychosocial disorders, such as anxiety (Cardona-Arias et al., 2015). Compared to populations with similar sociodemographic characteristics, university students have a higher risk of psychological disorders and experience approximately twice as much stress as reported by them (Silva & Figueiredo-Braga, 2018). However, they do not seek help because of society’s lack of understanding of mental health and, thus, fear of stigmatization (Marenco Escuderos et al., 2017). In this regard, recent research, such as that by Stangl et al. (2019), has highlighted that the stigma associated with anxiety disorders is particularly pronounced in university environments, which makes access to adequate treatments difficult and perpetuates emotional suffering.

Anxiety causes emotional disturbances in university students that have a great impact on cognitive functions, such as attention or memory, as well as academic performance and social and family relationships, as mentioned in the studies of (Freitas et al., 2023). One of the most frequent symptoms in university students with anxiety is insomnia, with the majority being female, and there are numerous studies that support the relationship between anxiety and female sex, youth (Knipe et al., 2018), and the condition of being a university student (Lun et al., 2018). Furthermore, studies such as Cabral et al. (2023) have found that excessive use of digital devices, especially in academic contexts, is associated with higher levels of insomnia and anxiety in university students, suggesting a bidirectional relationship between technology and mental health. The increase in the use of technology by young people today is undeniable, and the optimal use of technology can reduce anxiety levels in the university population, as evidenced in their study on technology applied to the treatment of anxiety problems. The types of ICTs that have been used in the treatment of anxiety problems are very diverse and include mobile assistants, internet-based treatment, different apps, and augmented reality as well as virtual reality (Regidor & Ausín, 2020). In particular, recent research, such as that of Huberty et al. (2019), has shown that mindfulness-based applications such as ‘Calm’ and ‘Headspace’ are effective in reducing anxiety symptoms in university students in Portugal, which reinforces the usefulness of these tools in educational contexts. Studies such as that of Forrester (2022) indicate that the use of emerging technologies in therapy as a therapeutic tool can be an element in reducing anxious symptomatology. Finally, Martínez Gimeno and Lopez Secanell (2022), in their study, show that mobile applications based on mindfulness are a means that can help to achieve the numerous psychological and physical benefits of mindfulness practices and, therefore, reduce anxiety, such as: ‘Petit BamBou’, ‘Ninja Focus’, or ‘Breathe, think and act’. However, it is crucial to note that excessive or inappropriate use of technology can have counterproductive effects, as pointed out by Mohd Saat et al. (2024), who found a positive correlation between screen time and anxiety levels in Spanish university students.

The main objective of this research is to analyze and determine the levels of state anxiety and risk anxiety in university students, assessing their prevalence and associated characteristics. It also seeks to examine the influence of the use of technologies in the regulation of anxiety in this population, identifying patterns of behavior and effectiveness in their application. In addition, this study aims to contrast the differences in anxiety levels according to variables such as gender and faculty in order to identify possible significant disparities. Finally, the aim is to compile and categorize the technological applications most used by students for anxiety management, providing a frame of reference for the preferred digital tools in this context.

## 2. Materials and Methods

### 2.1. Instrument

This study is descriptive in nature and employed a non-probabilistic purposive sampling. The main aim of this article is to assess both state anxiety (the transient emotional condition) and trait anxiety (a relatively stable anxious propensity) in students at the University of Burgos. In order to be able to answer the questionnaire, informed consent had to be accepted electronically as a prerequisite for completing the electronic version of the questionnaire. For data collection, the Spanish adaptation of the State-Trait Anxiety Inventory (STAI) (Buela-Casal et al., 2011) was used, as shown in Table 1. This version is composed of 40 items (i.e., 20 for each subscale). In the Spanish adaptation, the Likert-type response scale ranges from 0 to 3 points, unlike the original STAI, which ranges from 1 to 4 points (Spielberg, 1993). Although each subscale has a theoretical range of 20 to 80, the scores can be compared with those of the original scale by adding 20 to the scores obtained. According to Spielberg (1993), the totals for trait anxiety and state anxiety range from 0 to 60, and a higher score corresponds to a higher degree of anxiety. Scores ≥ 30 suggest moderate anxiety, and scores ≥ 45 suggest severe anxiety’, whereas scores ≤ 29 suggest low anxiety.

A series of questions were also added to the questionnaire with the aim of finding out whether the students at the University of Burgos used complementary applications to therapy to reduce anxiety:

- Would you consider using technology to try to reduce your anxiety levels?
- Have you used or do you use any mobile application to try to reduce your anxiety level?
- If you answered yes, please indicate on a scale of 0 to 3 how much it has contributed to reducing your anxiety level.
- Which of these anxiety-reducing apps do you know about (you can tick multiple answers)?

### 2.2. Participants

The student population of the University of Burgos is composed of a total of 8300 people; in order to reach a 95% sample reliability, at least 368 participants with a margin of error of 5% are required. The sample is composed of a total of 450 participants with a mean age of 26.46 years and a SD of 5.809. In terms of gender, the distribution of the sample is divided as follows: 70.4% of the sample are women, and the remaining 29.6% are men, as can be seen in Table 2. It should be noted that, regarding gender, the majority are women. This trend is notable in all faculties except for the Faculty of Science, where there are more men than women, and in the Higher Polytechnic School, where the proportion is more equal. All of this is in line with the population distribution of the University of Burgos, which gives the sample certain guarantees of representativeness when inferring the results found.

### 2.3. Procedure

**Figure 1:**
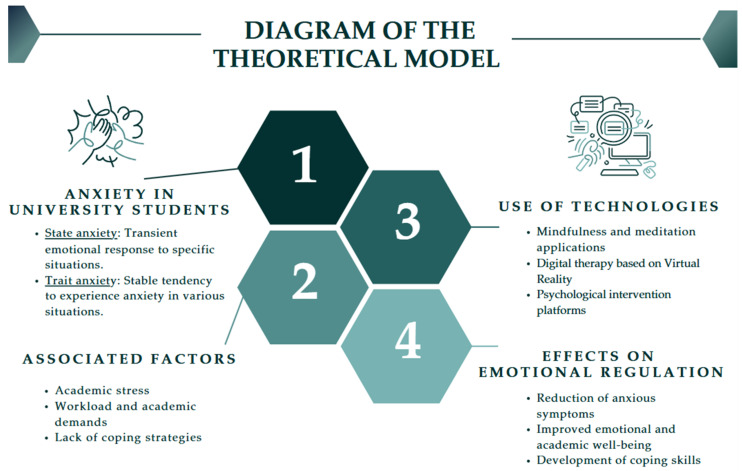
Diagram of the theoretical model.

Figure 1 below is a diagram that exemplifies the theoretical model of analysis that represents the relationship between anxiety in university students and the use of technologies for its management. It identifies the types of anxiety and the effects of technologies on emotion regulation.

## 3. Results

Table 3 shows the mean scores obtained, as well as the typical deviations of each of the 40 items that make up the questionnaire. The first 20 items correspond to the state anxiety category, and the next 20 to the trait anxiety category.

The mean and SD of the state anxiety category are 1.323 and 0.855, respectively. As can be seen in Table 3, there are differences between the various items that make up each of the two categories. In the aforementioned category, we find that the statement “I feel comfortable” is the item where the sample obtained the highest scores; however, within this category, item 14 for the statement “I feel very attached” obtains lower mean scores than the others.

In relation to the risk anxiety category, its mean and SD are 1.394 and 0.888, respectively. There are differences between the various items that make up the aforementioned category, with item 30 obtaining higher mean scores and item 35 corresponding to the statement “I feel sad” obtaining lower mean scores. Finally, the sum of both categories concludes that the total mean anxiety is 1.358 with a standard deviation of 0.872.

### 3.1. Category Anxiety Status with the Faculty Where They Study

The analysis of anxiety levels among university students, segmented by faculty, reveals a significant predominance of medium anxiety, as can be seen in Table 4. The Faculty of Education stands out notably, with 55.8% of its students presenting medium levels of anxiety and a small percentage (0.4%) presenting high anxiety, totaling 63.3% of the sample evaluated. This faculty shows the highest number of cases, suggesting specific factors that contribute to stress and anxiety in this student population; it should be taken into account that it is also the faculty from which the largest sample has been obtained. On the other hand, the sample obtained from the Faculty of Health Sciences also shows a considerable prevalence of medium anxiety, with 10.7% of the total sample, although no cases of high anxiety are recorded. Similarly, the Faculty of Law has 4.0% of students with medium anxiety and also shows no cases of high anxiety. However, the Faculty of Economics and Business Studies is unique in recording no cases of low or high anxiety, with 1.8% of the total sample and exclusively at the medium level of anxiety.

Finally, the Faculty of Science, with 2.9% of students experiencing medium anxiety, and the Polytechnic School, with 6.7%, also reflect this trend, with no cases of high anxiety. Finally, the Faculty of Humanities and Communication, although showing 5.6% of students with medium anxiety and one case of high anxiety (0.2%), remains within the general pattern. In summary, the majority of students assessed in all faculties experience medium anxiety (87.3% of the total), highlighting the need for specific interventions to manage this level of anxiety and prevent its escalation due to the fact that students face significant, although not extreme, levels of anxiety.

### 3.2. Category Anxiety Trait with the Faculty in Which They Study

Table 5 shows distinctive patterns of anxiety within each of the faculties. It is evident that medium trait anxiety was prevalent in most of the faculties assessed. Firstly, in the Faculty of Education, 53.6% of students were found to experience medium levels of anxiety, followed by the Faculty of Health Sciences with 10.7%. In addition, cases of high trait anxiety were identified in several faculties, most notably in Education (2.9%) and Humanities and Communication (0.9%). On the other hand, some faculties showed a lower incidence of anxiety, such as the Faculty of Business and Economics and Law, with lower percentages in both average trait anxiety, 1.6% and 3.8%, respectively. These results underline the variability of anxiety among university students according to their field of study, highlighting the need for tailored strategies to support mental health and well-being in diverse academic settings. Other faculties, such as the Faculty of Science (3.1%) and the Polytechnic School (6.4%), show mostly medium trait anxiety, with few cases of high trait anxiety. Finally, the Faculty of Humanities and Communication shows a distribution of medium trait anxiety (5.3%) and the same cases of low anxiety as high anxiety, with 0.9% in each of them. Overall, medium trait anxiety is the most common among all faculties, suggesting that most students face significant, though not extreme, levels of anxiety, totaling 84.4%.

### 3.3. Category Total Anxiety with the Faculty Where They Are Studying

Table 6 shows in detail the total anxiety levels of the students in the different faculties that make up the University of Burgos.

In the first place, we find the Higher Polytechnic School, with a total of 31 students evaluated, where 0.2% show low anxiety, while 6.4% show medium anxiety. No cases of high trait anxiety were identified in this faculty, contributing 6.9% of the total sample.

In second place, the Faculty of Science has 16 students evaluated; 0.7% show low anxiety, and 2.9% show medium anxiety. No cases of high trait anxiety were reported, representing 3.6% of the total.

In third place, the Faculty of Health Sciences shows a considerable distribution of anxiety, with a total of 59 students assessed. A total of 2.2% have low anxiety, and 10.9% have medium anxiety. No cases of high trait anxiety were recorded, contributing 13.1% of the total.

Next, the Faculty of Economics and Business Studies had eight students evaluated; 1.8% showed medium anxiety. No cases of low or high anxiety were identified, representing 1.8% of the total.

In the Faculty of Law, 19 students were evaluated, with 0.2% showing low anxiety and 4.0% showing medium anxiety. No cases of high anxiety were recorded, contributing 4.2% of the total.

The Faculty of Education stands out with a total of 285 students evaluated, showing 6.0% with low anxiety and a significant 56.9% with medium anxiety. Two students (0.4%) were identified as having high anxiety. Overall, this faculty represents 63.3% of the total assessed, showing the prevalence of medium anxiety among its students.

Finally, the Faculty of Humanities and Communication had 32 students evaluated; 0.7% show low anxiety, and 6.4% have medium anxiety. No cases of high anxiety were recorded, contributing 7.1% of the total.

In summary, most faculties show a significant prevalence of anxiety. These findings underline the importance of implementing effective anxiety management and support strategies tailored to promote student well-being in diverse academic environments.

Regarding the questions posed in relation to the use of applications to reduce anxiety levels, we can say that 61.7% consider that using technology to reduce anxiety levels is positive, and 21.2% use a mobile application to deal with anxiety levels. Likewise, students say that it helps them to relax somewhat (35.1%) and quite a lot (22.7), making up 57.7% of the total sample.

**Figure 2:**
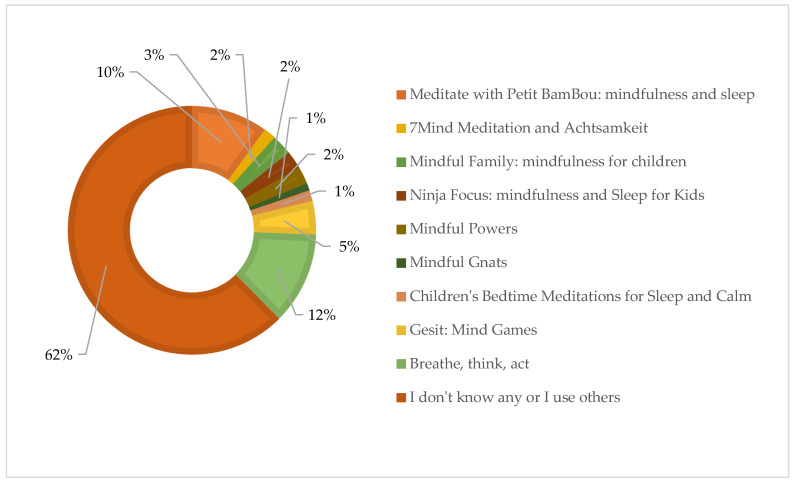
Distribution of the use and knowledge of various mindfulness and meditation applications among students.

Regarding the applications that students are most familiar with, Figure 2 shows the distribution of the use and knowledge of various mindfulness and meditation applications among the respondents. Most of the participants (76.1%) indicated that they do not know any of the listed apps or use other apps not mentioned in the survey. Among the specific apps, ‘Breathe, think, act’ is the best known, used by 14.6% of the respondents, followed by ‘Meditate with Petit BamBou: mindfulness for children’ with 12.2%. Other apps, such as ‘Gesti: mind games’ and ‘Mindful Family: mindfulness for kids & family’, are less well known, with 5.4% and 3.2% of users, respectively. The apps ‘Ninja Focus: mindfulness and Sleep’ (2.9%), ‘7Mind Meditation and Achtsamkeit für Kinder’ (2%), ‘Kids Meditations for Sleep and Calm’ (1.7%), ‘Mindful Powers’ (2.7%), and ‘Mindful Gnats’ (1.2%) have a lower usage rate. This graph shows that a large majority of respondents are not familiar with the mindfulness and meditation apps listed or prefer other apps not specified in the proposed list.

## 4. Discussion and Conclusions

The main objective of this study was to find out the level of anxiety in the university population of the University of Burgos, and after carrying out the analyses, it is clear that the students of the University of Burgos present average levels of anxiety in relation to the categories of state anxiety and risk anxiety. This fact is endorsed in studies such as those of Martin-Payo et al. (2021), which state that students present intermediate levels of anxiety with the level of risk anxiety predominating. It also shows that anxiety disorders are associated with the degree they are studying, gender, as well as the quality of sleep, and the different variables linked to the individual perception of the academic environment. Studies such as those by Martínez-Otero Pérez (2014) already showed that there were significant differences in prevalence and symptomatic patterns between the male and female genders. Women tend to experience anxiety disorders more frequently, which may be influenced by psychobiological and social factors. Likewise, other studies, such as those by Marquina-Luján et al. (2018), show a correlation between average anxiety levels and the tendency to procrastinate preferentially in the academic environment.

In contrast, studies such as that carried out by González-Aguilar (2021) show that the student population studying at university presents high levels of stress and anxiety, although it is true that anxiety on certain occasions has an activating function that facilitates the ability to respond, thus promoting the capacity for adaptation and preservation in the face of possible challenges posed by university life.

These studies reveal how technological tools, such as mobile applications and virtual reality, can be effective in reducing anxiety symptoms, improving accessibility to psychological treatments, and offering personalized options. Through the analysis of these studies, the potential advantages of integrating technology into therapeutic strategies are highlighted, opening new perspectives for the management of anxiety in diverse populations. Research such as that of Toledo del Castillo et al. (2019) shows how immersion in a virtual scenario helps to decrease anxious symptoms and psychological distress and induces relaxation through visual and auditory stimuli. For example, EMMA World, Engaging Media for Mental Health Applications, is a Virtual Reality program developed in a European Union project, designed with the aim of treating anxiety disorders in particular (Quero et al., 2017).

Similarly, Celleri and Garay (2021) state that treatment combined with mobile applications has shown success in improving adults with anxiety disorders. Apps that were designed particularly for anxiety disorders contain content such as cognitive restructuring and relaxation and breathing exercises.

The increase in these applications is such that there are various scales to assess the quality, effectiveness, reliability, and security of these applications, such as the Mobile App Rating Scale (MARS) (Martin-Payo et al., 2021) or the iSYScore index (Grau et al., 2016).

In their research, Martínez Gimeno and Lopez Secanell (2022) carried out a systematic review of mobile mindfulness applications for both Android and iOS to analyze their characteristics, quality, and suitability for educational contexts using scales such as MARS. In total, they obtained nine applications that exceeded the minimum acceptability score.

Likewise, technology can be taken as a tool that allows for predicting or indicating problems derived from anxiety in university students, as explained by Carranza Esteban et al. (2022), as an element of improvement of these levels. Studies such as that of Dias Lopes et al. (2020) indicate the need for preventive measures to minimize anxiety and help maintain the necessary levels of well-being during this phase of academic development and when building a professional career.

Table 7 presents a summary that synthesizes the relationship established by the self-researchers between anxiety levels and the use of mobile applications aimed at promoting anxiety management. The table details an analysis that provides a structured view of the interaction between app use and its impact on anxiety management, according to the perspective of the authors consulted.

Overall, this present study can be taken as a reference to promote a shift towards a perspective that includes the benefits of technology to improve mental health through mindfulness and meditation as a complementary tool to therapy.

It is important to note that the simple use of technology does not automatically lead to a reduction in anxiety levels. On the contrary, numerous studies have shown that excessive use of digital devices can increase levels of stress and anxiety, especially in academic contexts. For example, Twenge et al. (2018) found that excessive screen time is associated with higher levels of anxiety and depression in adolescents, suggesting that technology can have a negative impact on mental health when not properly regulated. Similarly, Liu et al. (2019) highlighted that reliance on digital devices in educational settings can lead to distraction and stress, affecting academic performance and emotional well-being. These findings reinforce the need to address technology use in a critical and balanced way, especially in contexts where its impact on mental health is significant.

Regarding the possible limitations of this study, it faces the challenge of not being able to include all students from the various faculties. This is because, in certain cases, some students do not attend the classes where they are asked to complete the questionnaire, do not check their email, or simply do not show a willingness to participate in the research. In addition, the inability to generalize its findings beyond the student population of the University of Burgos should be considered as a potential limitation of the study. This is due to the lack of a representative sample of students from other universities, which makes it difficult to establish general conclusions about the level of mindfulness.

This study faces the limitation of not including all students from the various faculties, which reduces the representativeness of the sample. In addition, the sampling used is non-probabilistic, which means that, although statistical significance is achieved, randomness in the selection of participants is not guaranteed. This restricts the generalizability of the findings to the entire university population and even within the institution where the study was conducted.

Regarding future lines of research, it would be interesting to make a national comparison of the different levels of anxiety that Spanish university students have and relate it to the benefits that technology brings to a reduction in anxiety. Similarly, it would be interesting to carry out a longitudinal study to follow the progress of these students and observe their evolution over the different courses. It would also be interesting to assess the anxiety levels of those students who use technology as a complementary tool compared to those who do not use it. Despite the limitations mentioned above and the possibility of delving deeper into this topic through different types of studies and carrying out different interventions with university students, an interesting picture is shown of the level of anxiety experienced by students at the University of Burgos.

## Figures and Tables

**Table 1:** Description of the State-Trait Anxiety Inventory Questionnaire (STAI) (Buela-Casal et al., 2011).

Category	Description	Items	Punctuation
Anxiety as a State (STAI-S)	It evaluates how a person feels at a specific and circumstantial moment.	1–20	Each item is scored on a scale of 0 to 3: 0 = almost never, 1 = sometimes, 2 = often, and 3 = almost always. The total score ranges from 20 to 80.
Anxiety as a Trait (STAI-T)	It assesses a person’s general willingness to feel anxious in everyday situations.	20–40	Each item is scored on a scale of 0 to 3: 0 = almost never, 1 = sometimes, 2 = often, and 3 = almost always. The total score ranges from 20 to 80.

**Table 2:** Description of participants by faculty.

Faculty	Man		Woman		Total	
	N	%	N	%	N	%
Higher Polytechnic Faculty	23	5.1%	8	1.8%	31	6.9%
Science Faculty	6	1.3%	10	2.2%	16	3.6%
Health Science Faculty	7	1.6%	52	11.6%	59	13.1%
Economic Science Faculty	3	0.7%	5	1.1%	8	1.8%
Law Faculty	8	1.8%	11	2.4%	19	4.2%
Education Faculty	72	16%	213	47.3%	285	63.3%
Humanities and Communication Faculty	14	3.1%	18	4.0%	32	7.1%
Total	133	29.6%	317	70.4%	450	100.0%

**Table 3:** Mean and standard deviation by items make up categories of the questionnaire.

Ítem	Media	DT
1. I feel calm	1.35	0.798
2. I feel safe	1.63	0.841
3. I’m tense	1.32	0.910
4. I am upset	0.88	0.863
5. I feel comfortable (I am at ease)	1.69	0.820
6. I feel upset	1.06	0.914
7. I am now worried about possible future misfortunes.	1.61	1.018
8. I feel rested	1.09	0.851
9. I feel distressed	1.12	0.911
10. I feel comfortable	1.42	0.731
11. I trust in myself	1.67	0.859
12. I feel nervous	1.37	0.937
13. I am restless	0.93	0.832
14. I feel very “tied” (like oppressed)	0.84	0.891
15. I’m relaxed	1.22	0.828
16. I feel satisfied	1.58	0.749
17. I’m worried	1.50	0.868
18. I feel dizzy and overexcited	0.91	0.877
19. I feel happy	1.63	0.796
20. I feel good right now	1.64	0.806
21. I feel good	1.69	0.813
22. I get tired quickly	1.49	0.933
23. I feel like crying	1.02	1.011
24. I would like to be as happy as others	1.18	1.054
25. I miss opportunities by not deciding soon	1.22	0.985
26. I feel rested	1.10	0.843
27. I am a calm, serene and peaceful person.	1.39	0.914
28. I see that the difficulties are piling up and I can’t handle them.	1.34	0.892
29. I worry too much about unimportant things	1.62	1.010
30. I’m happy	1.78	0.731
31. I tend to take things too seriously	1.72	0.814
32. I lack self-confidence	1.26	0.926
33. I feel safe	1.52	0.826
34. I don’t usually face crises or difficulties	0.98	0.858
35. I feel sad (melancholic)	0.92	0.844
36. I am satisfied	1.59	0.771
37. Unimportant thoughts haunt and bother me	1.40	0.852
38. I am so affected by disappointments that I cannot forget them.	1.36	0.989
39. I am a stable person	1.59	0.816
40. When I think about current issues and concerns I get tense and agitated.	1.71	0.882

**Table 4:** Category of anxiety status with the faculty in which they study.

Faculty	Anxiety Level State							
	Low		Medium		High		Total	
	N	%	N	%	N	%	N	%
Higher Polytechnic Faculty	1	0.2%	30	6.7%	0	0.0%	31	6.9%
Science Faculty	3	0.7%	13	2.9%	0	0.0%	16	3.6%
Health Science Faculty	11	2.4%	48	10.7%	0	0.0%	59	13.1%
Economic Science Faculty	0	0.0%	8	1.8%	0	0.0%	8	1.8%
Law Faculty	1	0.2%	18	4.0%	0	0.0%	19	4.2%
Education Faculty	32	7.1%	251	55.8%	2	0.4%	285	63.3%
Humanities and Communication Faculty	6	1.3%	25	5.6%	1	0.2%	32	7.1%
Total	54	11.9%	393	87.5%	3	0.6%	450	100.0%

**Table 5:** Category of anxiety trait with the faculty in which they study.

Faculty	Trait Anxiety Level							
	Low		Medium		High		Total	
	N	%	N	%	N	%	N	%
Higher Polytechnic Faculty	1	0.2%	29	6.4%	1	0.2%	31	6.9%
Science Faculty	2	0.4%	14	3.1%	0	0%	16	3.6%
Health Science Faculty	8	1.8%	48	10.7%	3	0.7%	59	13.1%
Economic Science Faculty	1	0.2%	7	1.6%	0	0.0%	8	1.8%
Law Faculty	1	0.2%	17	3.8%	1	0.2%	19	4.2%
Education Faculty	31	6.9%	241	53.6%	13	2.9%	285	63.3%
Humanities and Communication Faculty	4	0.9%	24	5.3%	4	0.9%	32	7.1%
Total	48	10.7%	380	84.4%	22	4.9%	450	100.0%

**Table 6:** Category of total anxiety with the faculty in which they study.

Faculty	Total Anxiety Level							
	Low		Medium		High		Total	
	N	%	N	%	N	%	N	%
Higher Polytechnic Faculty	1	0.2%	30	6.7%	0	0%	31	6.9%
Science Faculty	3	0.7%	13	2.9%	0	0%	16	3.6%
Health Science Faculty	10	2.2%	49	10.9%	0	0%	59	13.1%
Economic Science Faculty	0	0.0%	8	1.8%	0	0%	8	1.8%
Law Faculty	1	0.2%	18	4.0%	0	0%	19	4.2%
Education Faculty	27	6.0%	256	56.9%	2	0.4%	285	63.3%
Humanities and Communication Faculty	3	0.7%	29	6.4%	0	0%	32	7.1%
Total	45	10.0%	403	89.6%	2	0.4%	450	100.0%

**Table 7:** Findings between anxiety and technology.

Author (Year)	Anxiety-Technology Relationship	Main Findings
Forrester (2022)	Use of emerging technologies in therapy	Significantly reducing anxiety levels through digital interventions
Regidor and Ausín (2020)	Application of ICT in the treatment of anxiety	Diversity of tools (apps, VR) with positive effects on anxiety management
Martínez Gimeno and Lopez Secanell (2022)	Mindfulness through apps	Improved emotional stability and reduced anxiety
Toledo del Castillo et al. (2019)	Using virtual reality for relaxation	Induction of calm states and reduction in psychological distress
Celleri and Garay (2021)	Mobile apps for anxiety	Efficacy in adults with anxiety disorders, improvements in emotional well-being

## Data Availability

The original contributions presented in this study are included in the article. For further information, please contact the corresponding author.
